# Interplay of cooperative breeding and predation risk on egg allocation and reproductive output

**DOI:** 10.1093/beheco/arae010

**Published:** 2024-02-28

**Authors:** Rita Fortuna, Rita Covas, Pietro B D'Amelio, Liliana R Silva, Charline Parenteau, Louis Bliard, Fanny Rybak, Claire Doutrelant, Matthieu Paquet

**Affiliations:** CIBIO, Centro de Investigação em Biodiversidade e Recursos Genéticos, InBIO Laboratório Associado, Campus de Vairão, Universidade do Porto, 4485-661, Vairão, Portugal; Departamento de Biologia, Faculdade de Ciências, Universidade do Porto, 4099-002, Porto, Portugal; BIOPOLIS Program in Genomics, Biodiversity and Land Planning, CIBIO, Campus de Vairão, 4485-661, Vairão, Portugal; Centre for Biodiversity Dynamics, Institutt for Biologi, NTNU, 7491, Trondheim, Norway; CIBIO, Centro de Investigação em Biodiversidade e Recursos Genéticos, InBIO Laboratório Associado, Campus de Vairão, Universidade do Porto, 4485-661, Vairão, Portugal; BIOPOLIS Program in Genomics, Biodiversity and Land Planning, CIBIO, Campus de Vairão, 4485-661, Vairão, Portugal; FitzPatrick Institute, DST-NRF Centre of Excellence, University of Cape Town, 7701, Cape Town, South Africa; CEFE, Univ Montpellier, CNRS, EPHE, IRD, 34293, Montpellier, France; Université Paris-Saclay, CNRS, Institut des Neurosciences Paris-Saclay, 91400, Saclay, France; CIBIO, Centro de Investigação em Biodiversidade e Recursos Genéticos, InBIO Laboratório Associado, Campus de Vairão, Universidade do Porto, 4485-661, Vairão, Portugal; BIOPOLIS Program in Genomics, Biodiversity and Land Planning, CIBIO, Campus de Vairão, 4485-661, Vairão, Portugal; Centre d’Etudes Biologiques de Chizé, CNRS-La Rochelle Université, 79360, Villiers-en-Bois, France; Department of Evolutionary Biology & Environmental Studies, University of Zurich, Winterthurerstrasse 190, CH-8057 Zurich, Switzerland; Université Paris-Saclay, CNRS, Institut des Neurosciences Paris-Saclay, 91400, Saclay, France; FitzPatrick Institute, DST-NRF Centre of Excellence, University of Cape Town, 7701, Cape Town, South Africa; CEFE, Univ Montpellier, CNRS, EPHE, IRD, 34293, Montpellier, France; Institute of Mathematics of Bordeaux, University of Bordeaux, CNRS, Bordeaux INP, 33405, Talence, France; Theoretical and Experimental Ecology Station (SETE), CNRS, 09200, Moulis, France

**Keywords:** clutch size, cooperative breeding, corticosterone, egg mass, group size, maternal allocation, playback experiment, predation risk, reproductive output, yolk mass

## Abstract

Predation risk can influence behavior, reproductive investment, and, ultimately, individuals’ fitness. In high-risk environments, females often reduce allocation to reproduction, which can affect offspring phenotype and breeding success. In cooperative breeders, helpers contribute to feed the offspring, and groups often live and forage together. Helpers can, therefore, improve reproductive success, but also influence breeders’ condition, stress levels and predation risk. Yet, whether helper presence can buffer the effects of predation risk on maternal reproductive allocation remains unstudied. Here, we used the cooperatively breeding sociable weaver *Philetairus socius* to test the interactive effects of predation risk and breeding group size on maternal allocation to clutch size, egg mass, yolk mass, and yolk corticosterone. We increased perceived predation risk before egg laying using playbacks of the adults’ main predator, gabar goshawk (*Micronisus gabar*). We also tested the interactive effects of group size and prenatal predator playbacks on offspring hatching and fledging probability. Predator-exposed females laid eggs with 4% lighter yolks, but predator-calls’ exposure did not clearly affect clutch size, egg mass, or egg corticosterone levels. Playback-treatment effects on yolk mass were independent of group size, suggesting that helpers’ presence did not mitigate predation risk effects on maternal allocation. Although predator-induced reductions in yolk mass may decrease nutrient availability to offspring, potentially affecting their survival, playback-treatment effects on hatching and fledging success were not evident. The interplay between helper presence and predator effects on maternal reproductive investment is still an overlooked area of life history and physiological evolutionary trade-offs that requires further studies.

## Teaser

This study addresses the current knowledge gap on how the reproductive allocation of egglaying females exposed to predator cues differs in cooperative breeding systems where individuals breed in groups when breeding females have a variable number of helpers Results suggest that predatorcall exposure affected maternal allocation to offspring, via reductions in yolk mass, which is the major source of nutrients in the egg, but breeding with more helpers did not appear to buffer these predatorinduced effects.

## INTRODUCTION

Predation can affect individuals’ fitness and prey population dynamics (McNamara and Houston 1987; Hanski et al. 1993; Krebs et al. 1995), not only by causing direct mortality but also through the perceived risk (or “fear”) of predators, which can affect the behavior, reproductive strategies, and social interactions of prey (Lima 1998; Zanette et al. 2011; Dudeck et al. 2018; Allen et al. 2022). Antipredator behavioral responses may entail substantial fitness costs (Creel and Christianson 2008), because the need to avoid immediate predation may force individuals to decrease or change foraging patterns and/or breeding investment (Lima 1998, 2009; Cresswell 2008; LaManna and Martin 2016). For example, under high predation pressure, individuals were found to trade-off the time spent foraging with vigilance behavior (Sansom et al. 2008; Creel et al. 2014), and decrease food intake and/or offspring provisioning rates (Ghalambor and Martin 2000; Tilgar et al. 2011), sometimes at an evident cost to the young (Scheuerlein and Gwinner 2006; Dudeck et al. 2018). Moreover, predators’ presence or perception can lead to physiological stress responses and to chronic levels of circulating glucocorticoids (Clinchy et al. 2004, 2013) which may, in turn, compromise the immune and reproductive systems (Sapolsky 1992; Boonstra et al. 1998; Scheuerlein et al. 2001; Navarro et al. 2004). Along with its behavioral and physiological effects, predator presence may influence the amount of energy and resources individuals allocate to reproduction, particularly for females that produce eggs or carry the developing embryos.

According to life-history theory, individuals should balance investment in current reproduction against survival and future breeding opportunities (Stearns 1992; Roff 1993). Under high predation risk, females with a low probability of future breeding might therefore increase investment in current reproduction, while females with high future breeding probability might reduce current investment, if this minimizes exposure to predators and consequently increases their survival (Clark 1994). Accordingly, in some bird species, females exposed to predator-call playbacks were found to lay less eggs per clutch, sometimes producing heavier eggs instead (e.g., in Eastern bluebirds *Sialia sialis*: Hua et al. 2014; in song sparrows *Melospiza melodia*: Zanette et al. 2011; but see Coslovsky and Richner 2011), as producing less and larger offspring may allow them to reduce the risk of being depredated while providing postnatal care (Lima 1987, 2009). Besides, predator presence during egg formation can influence the composition of the eggs laid (Saino et al. 2005; Morosinotto et al. 2013). While evidence for variation in egg nutritional reserves is still limited (Coslovsky et al. 2012; Morosinotto et al. 2013), maternal transfer of hormones to eggs has been found to vary with predator presence, with females under high predation risk laying eggs with higher corticosterone levels and lower testosterone levels (Coslovsky et al. 2012; Saino et al. 2005; but see Morosinotto et al. 2016). This can have detrimental effects on offspring growth (Saino et al. 2005; Henriksen et al. 2011a; Pitk et al. 2012), even though it has also been shown to correlate with increased offspring flight performance (Chin et al. 2009), potentially enabling offspring to better evade predators in high-risk environments (Mousseau and Fox 1998; Storm and Lima 2010; Coslovsky and Richner 2011; Sheriff and Love 2013; Morales et al. 2018). Ultimately, the effects of higher perceived predation risk on reproductive investment may lead to the production of less successful offspring (Zanette et al. 2011; Hua et al. 2014), sometimes causing substantial reductions in annual reproductive output (Zanette et al. 2011). However, there appears to be no general rule among species on whether and how predation risk influences reproductive allocation and subsequent offspring survival (Fontaine and Martin 2006; Lima 2009; LaManna and Martin 2016). This might be due to predation risk effects being often studied in a mixed context of adult and offspring predators (Zanette et al. 2011), or only in relation to offspring predation risk (Fontaine and Martin 2006; Lima 2009; Martin and Briskie 2009; LaManna and Martin 2016), while predator presence may lead to different responses depending on whether it threatens the survival of the offspring or the breeder (Hua et al. 2014). Furthermore, previous work suggests that antipredator responses may be species- and context-specific (e.g., vary with reproductive tactics and breeding environment; LaManna and Martin 2016) and therefore better understood when relevant variables related to their life history and ecological context are considered.

In many species, there is evidence that being part of a group can reduce the chances of being depredated (Hamilton 1971; Bertram 1978; Foster and Treherne 1981; Rasa 1989). In cooperatively breeding species, breeders and helpers form breeding groups that collectively provide care to the young, and having helpers generally improves breeders’ reproductive output (Downing et al. 2020) and survival (Downing et al. 2021). Helpers may also contribute to other group activities, such as protecting offspring from predators, and they often forage and live with the breeders before and during reproduction (Dickinson and Koenig 2016). There are several reasons to hypothesize that breeders with more helpers may be at an advantage in high predation-risk environments. First, foraging in larger groups may lead to reduced predation risk through dilution effects, predator confusion, or higher efficacy at detecting predators (Hamilton 1971; Vine 1971; Lima 1995; Sorato et al. 2012). For instance, in the cooperatively breeding chestnut-crowned babbler *Pomatostomus ruficeps*, larger groups had a lower probability of being attacked by avian predators, and individual group members experienced lower predation risk (Sorato et al. 2012). Moreover, parents can often reduce feeding rates when they have helpers (i.e., “load-lightening”; Brown 1978; Crick 1992; Hatchwell 1999), and may, therefore, reduce their own exposure to predators by reducing the number of entries/exits from the nest, and time spent foraging. Being part of a larger group may also improve females’ condition before reproduction, as individuals that forage in groups can have higher foraging efficiency (Bertram 1978; Bednarz 1988), and individuals can gain thermoregulation benefits from huddling or roosting together (du Plessis and Williams 1994; Hatchwell et al. 2009; Paquet et al. 2016). Helper number might therefore influence breeding females’ behavior and stress levels in response to predation risk, reduce their energy expenditure and predator exposure when breeding, and can therefore be predicted to mitigate predator-induced changes in reproductive allocation. Although considerable attention has been paid to the independent effects of predators (see above) and helpers on maternal allocation strategies and reproductive output (Dixit et al. 2017; Downing et al. 2020; Fortuna et al. 2021), interactive effects of helper number and predation risk on maternal allocation strategies have not been, to our knowledge, explored.

In this study, we experimentally increase perceived predator presence, by playing back calls of a predator of adults and test how this affects maternal allocation—clutch size, egg mass, yolk mass, and yolk corticosterone—and reproductive output—hatching and fledging success. The study was conducted on the colonial cooperatively breeding sociable weaver (*Philetairus socius*), with the aim of assessing how breeding group sizes may modulate maternal allocation responses to predation risk. Sociable weavers are facultative cooperative breeders and helpers assist with feeding the offspring and with nest building and protection (Maclean 1973a; Ferreira 2015). Most helpers are previous offspring of the breeders (Covas et al. 2006; Fortuna et al. 2022) and roost with the breeding pairs before breeding starts (Paquet et al. 2016). Sociable weaver colonies generally divide in smaller flocks to forage (Lloyd et al. 2017), but individuals within breeding groups formed by breeders and helpers show the strongest social associations while feeding (Ferreira et al. 2020). Moreover, parents visit nests less often when breeding in larger groups (Covas et al. 2008), suggesting that having more helpers may reduce breeders’ exposure to predators during nestling rearing.

Given existing theory and the life history of sociable weavers, which are a multi-brooded species with relatively high adult survival (Paquet et al. 2015; Fortuna et al. 2021), predator-exposed females are predicted to prioritize their survival and future breeding chances and therefore reduce the clutch size, or lay lighter eggs with lighter yolks, than females in the control treatment (or produce less but heavier eggs, see Zanette et al. 2011). Females in smaller breeding groups are expected to show a stronger reduction in maternal allocation, as these will incur higher predation risk by feeding the offspring more often than females breeding with more helpers, and may benefit less from group foraging or communal roosting effects on body condition. In addition, predator-exposed females in smaller breeding groups are expected to experience the highest stress levels and, thus, to lay eggs with higher corticosterone concentration than predator-exposed females with more helpers, or females in the control treatment. Consequently, for reproductive output, we predict lower hatching and fledging success in nests where females were exposed to predator-call playbacks at laying but weaker predator-treatment effects for females breeding with more helpers.

## METHODS

### Study system

This experiment was performed from January to March 2020 in a population of sociable weavers, which is part of a long-term study at Benfontein Nature Reserve, Northern Cape Province of South Africa (28°520 S, 24°500E). The study area is located at the southeastern edge of the distribution range of the species and consists of an open *Acacia erioloba* savanna environment.

Sociable weavers build communal nests or “colonies” containing numerous independent chambers where breeding pairs or groups raise the chicks, and individuals show communal roosting throughout the year (Maclean 1973b). These colonies were captured annually to obtain information on colony size, mark individuals with a unique numbered metal ring and color combination, measure and weigh birds, and obtain blood samples (for genetic analyses). The color rings allow posterior visual identification of individuals in video recordings. Helpers assist primarily by provisioning food to offspring but can also mob nest predators (Rat 2015) and assist with nest building and sanitation (Ferreira 2015).

### Manipulation of perceived predation risk

We simulated the presence of one of the main predators of sociable weaver adults, the gabar goshawk (Maclean 1973d). Gabar goshawks can seize both adult and post-fledgling juvenile sociable weavers in flight (Maclean 1973d) and are often heard and seen in the study area, including observations of predation events (authors pers. obs.; Brown et al. 2003). Acoustic cues indicating or simulating the presence of this predator cause sociable weavers to seek refuge in trees or hide in the colony’s nest chambers, and birds often go silent when inside the nests (author’s personal observation). Experimental playbacks of predator calls have been found to elicit antipredator responses in numerous speciesspecies (Zanette et al. 2011; Hua et al. 2014; Abbey-Lee and Dingemanse 2019). We thus used playbacks of gabar goshawk calls, who are vocal birds during display (Kemp and Kirwan 2020), to simulate the regular presence of these goshawks near the breeding colonies before egg laying. In control colonies, we played-back calls of ringed-necked dove (*Streptopelia capicola*), a harmless bird for sociable weavers that is commonly found around the colonies.

To create the playback tracks, we extracted recordings of four adult gabar goshawks and four ringed-necked doves from the online repository *xeno-canto* (www.xeno-canto.org; file sources and credits can be found in doi:10.5061/dryad.rjdfn2zkk). Recordings from xeno-canto were processed in Audacity v.2.3.2 (available at https://www.audacityteam.org/; Audacity 2019) to minimize background noise, by applying high or low pass filters and noise reduction according to the requirements of each recording. Each playback session lasted 2 h at a ratio of sound to silence of 1:1.5 (i.e., 48 min of signal and 72 min of silence in 2 h; see Zanette et al. 2011). We built four 2-h playback tracks of goshawks and four 2-h playback tracks of doves (each including calls from just one individual). For this, we assembled predator calls in 1-min bouts, which was the natural maximum duration of an adult goshawk call sampled from the files collected in the online repository. Within each 1-min bout, we combined six calls lasting approximately 3 s and belonging to the same individual, with a distribution of silences between calls that were randomly sampled from the natural distribution of silences estimated between 13 bouts of calls of 5 different individuals (range from 2 to 18 s, mean ± standard deviation or SD = 6.8 ± 4.6 s). Each bout was faded in and out. One-minute bouts were then merged to create a 2-h playback track, with each 1-min separated from the following one by silence intervals of different durations (30 s, 1 min, 1 min and a half, and 3 min), to minimize predictability of calls. The order of appearance of the four silence intervals between the 1-min bouts was randomized each 10 min. Two-hour tracks were played back at a volume of approximately 90dB at 1 m distance from the speaker (Abbey-Lee and Dingemanse 2019). Control tracks were built using the same design as predator tracks.

We conducted this experiment in six study colonies, splitting them into predator and control treatments (three colonies each). The predator and control-treatment colonies were chosen based on similar colony sizes, that is, the total number of birds captured that season across the three colonies (163 birds in the predator-treatment colonies and 172 birds in the control-treatment colonies) and a mixed distribution across the study area to minimize effects of spatial autocorrelation (Supplementary Figure S1).

The experiment started on 26 January 2020 and stopped when the first chick of each colony hatched (corresponding to 14 March 2020 for the last colony; mean treatment duration = 41 days for predator and 39 days for control treatment; minimum treatment duration = 28 days). To minimize the risk of habituation, playbacks were performed every second day at each colony. We used an Anker Soundcore Motion+ speaker (Soundcore, United States, https://www.soundcore.com/) placed inside a cardboard box wrapped in plastic. The speaker box was installed on a tree close to each colony attached to the tree branches with ropes, approximately 1.5 m high (distance from tree to colony varied between 28 and 40 m; average distance in predator-treatment colonies was 35 m and in control-treatment colonies was 33 m). The playbacks automatically started 20 min before sunrise by programming a single-board computer (Raspberry pi 3, model B, Raspberry Pi Foundation, United Kingdom, https://www.raspberrypi.org/) attached to a digital-to-analog sound converter board (PIFI DAC + v2.0, China, https://www.kubii.fr/) connected to the speaker. Both the speaker and the single-board computer were powered by portable batteries. The computer and battery were kept in a plastic camouflaged box on the ground next to the tree bearing the speaker box. All material was moved every day from three colonies to the other three, except the cardboard boxes that were kept at the colonies throughout the playback experiment. Every predator-treatment colony was exposed to all four playback sessions of predator calls in different days, and every control-treatment colony was exposed to all four playback sessions of dove calls in different days. Two people verified that playbacks at one colony could not be heard from other experimental colonies.

To track possible automatic playback failures due to batteries/cables malfunctions caused by wildlife or weather conditions, we additionally installed an audio recorder with a microphone to detect the playback recordings. When playbacks failed to start at sunrise, or heavy rain was predicted during nighttime or at sunrise, playbacks were performed later in the scheduled day during late morning/early afternoon (34 out of 115 playback sessions), except when heavy rains did not cease in which case playback sessions were canceled (re-starting 2 days after). In total, 115 two-hour playback sessions were performed, 57 in control colonies and 58 in treatment colonies.

### Breeding monitoring procedures

From the start of the experiment, we monitored nests every 2 days at the six colonies. After the playback was over each morning, nests were checked for new clutches. Sociable weavers usually lay one egg per day in the morning and start incubating before the clutch is complete (Maclean 1973c). When the first egg of a clutch was found, the nest was visited every day to mark new eggs with a soft blunt pencil and weigh them to the nearest 0.001 g with a digital Pesola balance. Weighing of all eggs was done at the third laying day, since most sociable weaver clutches have three eggs (Fortuna et al. 2021). Furthermore, the third-laid egg of each clutch was collected after weighing and kept frozen at −20 °C to measure yolk mass and corticosterone concentration. For ethical and practical reasons, we collected only one egg. Specifically, collecting the third egg minimized the risk of collecting eggs while the laying process was occurring, with the additional advantage that the third egg was collected on the same morning it was laid, and thus little incubated. Nests were also checked on the next day to weigh possible fourth eggs. Clutches of five eggs are very rare (Fortuna et al. 2021) but were also weighed when found (*N* = 1). We stopped weighing and collecting eggs from clutches that were laid one day after the last playbacks stopped at each colony. The first clutch was sampled after 9 playback sessions in the control colonies and after 10 playback sessions in the predator-treatment colonies (i.e., ca. 20 days after the first playback session), whereas the last clutch was sampled after 21 or 19 playback sessions in the control and predator treatments, respectively (mean = 15 playback sessions before laying, for both treatments). This exposure should allow detection of treatment effects on maternal egg allocation, since yolks start forming only ca. 4–5 days before laying in passerines (Walsberg 1983; Gill 1995; Perrins 1996). Analogous acoustic experiments with similar playback exposure time and frequency have reported strong predator treatment effects on egg allocation and offspring survival (Zanette et al. 2011; Hua et al. 2014).

Nests were monitored until hatching, and when the first nestling was 9 days old, nestlings were ringed with a unique numbered aluminum ring, weighed, and a blood sample was taken. When the first chick was 16–18 days of age (the last days’ nests can be visited without increasing the chances of inducing fledging), nests were visited to assess brood survival, and chicks that were alive were considered as having fledged. The fate and fate date of each egg and non-fledged chick were also registered.

We conducted surveys at the colonies in the mornings where playbacks took place to assess the presence of gabar goshawks during the experiment. For 5 min, all gabar goshawk sightings or calls were recorded by inspecting all trees and skyline around the colonies. Gabar goshawks were detected in 3 of 165 surveys, twice near a treatment colony and once near a control colony, indicating that our playbacks did not seem to attract this predator to the colonies. All encounters with gabar goshawks near the colonies at any moment during the experimental period (ca. 2 months) were also recorded. Gabar goshawks were spotted once perching at a predation-treatment colony tree and once chasing a weaver at a control colony.

### Yolk mass and corticosterone measurements

Yolks were separated from the albumen while defrosting and wet yolk was weighed at the nearest 0.001 g. Corticosterone was assayed via radioimmunoassay in two runs. In detail, 100 mg of each sample were homogenized in 1 mL of distilled water and three to four glass beads, using a vortex. Steroids were extracted by adding 3 mL of diethyl-ether to 300 μL of the mixture, vortexing, and centrifuging (5 min at 2000 rpm, at 4 °C). The diethyl-ether phase containing corticosterone was decanted and poured off after snap freezing the tube in an alcohol bath at minus 40 °C. This was done twice for each yolk, and the solvent was then evaporated at 37 °C. The dried extracts were re-dissolved in 800 μL of phosphate 0.01 M pH 7.4 buffer, and the hormone was assayed in duplicate. 100 μL of extract were incubated overnight at 4^o^C with 4000 cpm of the appropriate H3-steroid (Perkin Elmer, US) and polyclonal rabbit antiserum. Anti-corticosterone antiserum was supplied by Merck. The bound fraction was then separated from free fraction by the addition of dextran-coated charcoal, and activity was counted on a tri-carb 2810 TR scintillation counter (Perkin Elmer, US). Tests were performed to validate the corticosterone assay on egg yolk samples. Yolk extracts were serially diluted in the assay buffer, and their displacement curves were parallel to the standard curve. Inter- and intra-assay variations were respectively 10.42% and 8.91%. Corticosterone’s lowest detectable concentration in yolk extracts was 56.3 pg/mL. The assay specificity was evaluated by spiking extracts, and recovery was 113.3%. Cross-reactions of corticosterone antiserum were as follows: 11-dehydrocosticosterone (0.67%), deoxycorticosterone (1.5%), 18–hydroxy-deoxycorticosterone (<0.01%), cortisone (<0.01%), progesterone (0.004%), and aldosterone (0.2%).

### Group size and breeder identity

Nests were video recorded to obtain information on the breeding group sizes and the identity of the breeders and helpers visiting the nests based on their color rings (Silva et al. 2018). Video cameras were placed on tripods under the colonies focusing on the entry of the target nests (Silva et al. 2018). During the incubation period, nest visits were recorded for a minimum of 60 min and on average for 3 h (mean ± SD = 195 ± 95 min), in two different days, once between second and fourth day of incubation and another between 8–10 days after the laying of the first egg. During the nestling period, nest visits were recorded for ca. 3 h (mean ± SD = 174 ± 59 min) on two different days, between days 8–12 and days 9–15 after hatching of the first chick.

Breeding group size could only be estimated in 21 out of 90 nests (12 in the control treatment and 9 in the predator treatment) due to nestlings’ mortality before reaching 8–12 days of age (the mean group size ± SD in this sample was 3.0 ± 1.1, *N* = 21). Since helpers visit the nests during incubation, we used instead the number of birds seen during this period as the group size measure for further analyses, which could be obtained for 76 nests. In this latter sample, there were 38 nests in each treatment. The mean incubation group size ± SD was 3.379 ± 1.219 (Spearman’s rank correlation coefficient between incubation group size and nestling period’s group size = 0.58; *P* = 0.007; *N* = 21 comparisons). All individuals seen visiting, building, and/or feeding at the nest were considered part of the group, except individuals that were attacked by a member of the group when trying to enter the nests.

After excluding nests with missing group size information, the available sample sizes to estimate treatment effects on clutch size, egg mass, and yolk mass/corticosterone levels were respectively 84 clutches, 257 eggs weighed from 77 clutches, and 68 third-laid eggs. To estimate effects on egg hatching success, we used 141 eggs from 55 clutches, and for fledging success from hatching we included 56 nestlings from 26 clutches.

### Statistical analyses

To test how experimentally increased perceived predation risk affected maternal allocation strategies, and whether this effect was conditional on breeding group sizes, we built four linear mixed models including each measure—clutch size, egg mass, yolk mass, and yolk corticosterone—as a dependent variable and treatment and group size in a two-way interaction and as single independent variables. Treatment was included as a binary factor (0 for control, 1 for predator-call playbacks). All analyses were conducted using the R software v.4.0.4 (R Core Team 2022). All models were fitted assuming a normal error distribution by restricted maximum likelihood in “lme4” (Bates et al. 2015). Clutch size models were under-dispersed using a Poisson error, and therefore a normal error distribution was also assumed (see Fortuna et al. 2021).

In the egg mass model, we included clutch identity, nest identity (in six nests, more than one clutch was laid, representing 20 additional eggs laid in repeated nests), and colony identity as random terms. For the models analyzing clutch size, yolk mass, and yolk corticosterone, there was only one value per clutch; hence, we only included clutch identity and colony identity as random terms. Additionally, in the yolk mass model, we accounted for egg mass (mean ± SD = 2.57 ± 0.21) as a predictor of yolk mass but also tested if results were the same when removing this variable (i.e., absolute changes in yolk mass; Paquet et al. 2013; Fortuna et al. 2023). Similarly, effects on corticosterone concentration were tested when including egg mass and yolk mass (mean ± SD = 0.51 ± 0.06) in the model, thus testing for an absolute change in corticosterone levels.

To test effects on egg hatching probability and fledging probability from hatching (as binary variables, 0 for failure and 1 for success), we built two generalized linear mixed models assuming a “binomial” distribution with logit link function, and similar fixed and random effects structures as described before, including both nest and clutch identity for the hatching probability model but only nest identity in the fledging probability model (only one clutch per nest hatched). Only nests where the third egg has been collected were considered to build the datasets for these models, and collected eggs were excluded and thus not considered as having failed to hatch/fledge.

Numerical predictor variables were divided by their standard deviation and centered by subtracting their mean, to allow interpretation of main effects when these are included in an interaction (Schielzeth 2010). Collinearity among predictors was assessed by calculating Spearman rank correlation coefficients (all < 0.4). Model diagnostics were assessed using the “performance” (Lüdecke et al. 2021) and “DHARMa” packages (Hartig 2021). Type-II Wald χ^2^ tests were used to estimate P values. No model reduction was performed. Effects were considered statistically significant when confidence intervals did not overlap 0, and *P* values were lower than 0.05. Results are presented as estimated means [and 95% confidence intervals]. Plots show raw data and model predicted means and 95% confidence intervals estimated using the “ggeffects” package (Lüdecke 2018; see code for further details).

## RESULTS

### Maternal egg allocation

**Figure 1:**
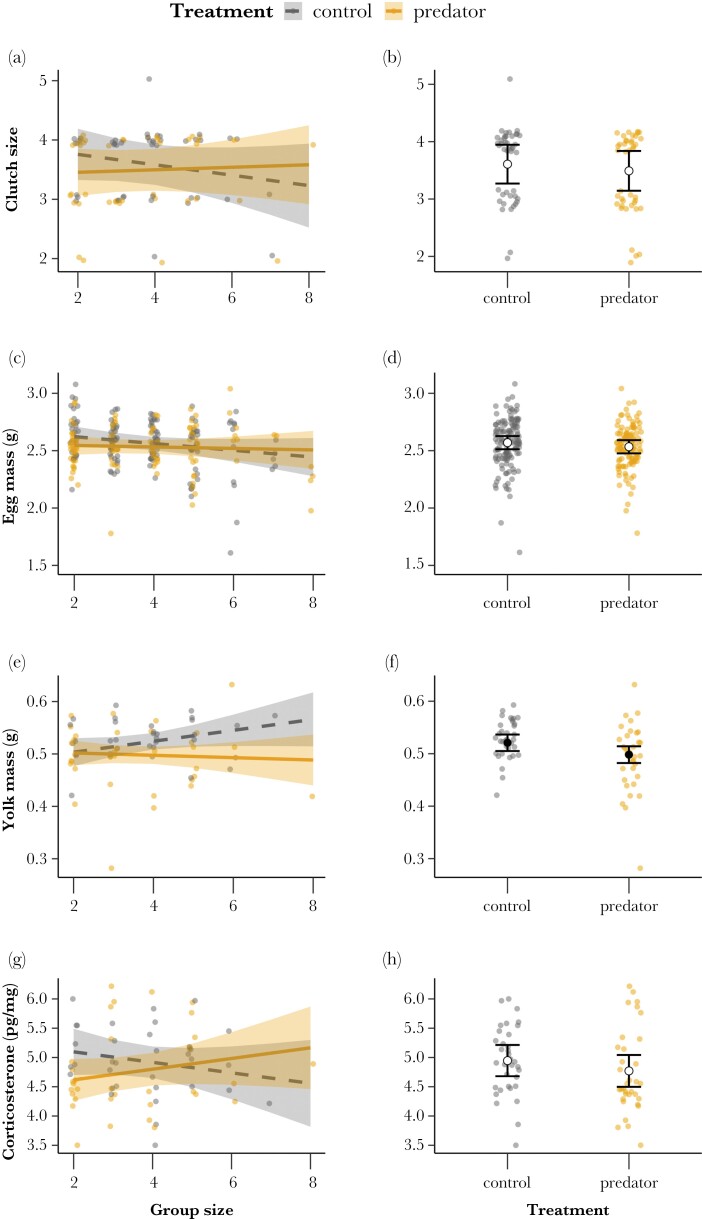
Predicted effects of the interaction between treatment and group size, and treatment as a single effect, respectively, on clutch size (a, b), egg mass (c, d) yolk mass (e, f), and corticosterone (g, h). Control playback is indicated by the gray points and dashed line, and predator-call playback by the orange points and solid line. Lines (left) or circles (right) show mean predicted effects and bands (left) or bars (right) show 95% confidence intervals. Full circles represent statistically supported differences between treatments. Points show observed values.

We did not detect main effects of the playback-treatment on clutch size (−0.117 [−0.568, 0.346]; *P* = 0.627; Figure 1B), egg mass (−0.036 g [−0.123, 0.044]; *P* = 0.379; Figure 1D) and yolk corticosterone levels (−0.176 pg/mg [−0.501, 0.106]; *P* = 0.362; Figure 1H), and there were also no detectable effects of the interaction between treatment and group size for these three maternal allocation proxies (clutch size: 0.158 [−0.125, 0.434]; *P* = 0.267; Figure 1A; Supplementary Table S1; egg mass: 0.034 [−0.041, 0.109]; *P* = 0.377; Figure 1C; Supplementary Table S2; yolk corticosterone: 0.26 [−0.069, 0.546]; *P* = 0.1; Figure 1G; Supplementary Table S5). Qualitatively similar results were obtained for corticosterone absolute changes (i.e., including egg mass and yolk mass in the model; see Supplementary Table S6).

On the other hand, females in the predator treatment laid eggs with lighter yolks than control-treatment females (−0.023 g [−0.044, −0.001]; *P* = 0.036) with a predicted yolk mass for third-laid eggs in control nests of 0.52 g [0.51, 0.54] and of 0.50 g [0.48, 0.51] in predator-treatment nests (Figure 1F; Supplementary Table S3). A significant effect of similar strength was still observed when testing for absolute yolk mass changes (i.e., excluding egg mass as covariate; see Supplementary Table S4). We furthermore tested whether this difference between treatments was driven by one low yolk mass value estimated in predator-treatment colonies (0.282 g; see Figure 1F), but the treatment effect size was of similar magnitude after excluding this data point, and the effect was still statistically significant. Nevertheless, we did not detect a statistically clear effect of the interaction between group size and treatment on yolk mass (−0.018 [−0.040, 0.003]; *P* = 0.108; Supplementary Table S3), finding only a tendency for an increase in yolk mass with number of helpers for control-treatment females but not for predator-exposed females (Figure 1E).

No evident main effects of group size were detected for clutch size (−0.128 [−0.335, 0.088]; *P* = 0.611; Supplementary Table S1), egg mass (−0.044 [−0.099, 0.011]; *P* = 0.184; Supplementary Table S2), yolk mass of the third-laid egg (0.015 [−0.001, 0.031]; *P* = 0.392; Supplementary Table S3) or corticosterone concentration (−0.129 [−0.34, 0.118]; *P* = 0.845; Supplementary Table S5).

### Offspring survival: hatching and fledging success

**Figure 2:**
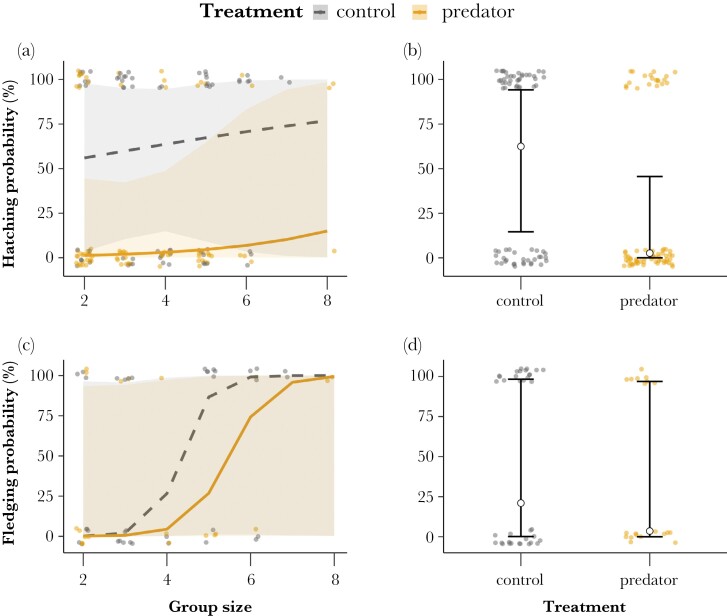
Predicted effects of the interaction between treatment and group size, and treatment as a single effect, respectively, on egg hatching probability (a, b) and chick fledging probability (c, d). Control playback is indicated by the gray points and dashed line, and predator-call playback by the orange points and solid line. Lines (left) or circles (right) show mean predicted effects and bands (left) or bars (right) show 95% confidence intervals. Full circles represent statistically supported differences between treatments. Points show observations of hatching/fledging failure (0%) or success (100%).

Eggs laid by females exposed to the predator-call playback tended to have lower chances of hatching than eggs laid by females in the control treatment (−4.115 [−8.497, 0.267]; *P* = 0.066; Supplementary Table S7). The predicted mean egg-hatching probability decreased from 62% [15, 94] in the control treatment to 3% [0, 46] in the predator treatment (Figure 2B), but with wide and overlapping confidence intervals (Figure 2A,B). Playback-treatment effects on hatching were not found to interact with females’ group size (0.423 [−2.611, 3.457]; *P* = 0.785; Supplementary Table S7), and hatching probability did not detectably vary with group size as a single term (0.249 [−2.199, 2.697]; *P* = 0.492; Supplementary Table S7).

Fledging probability from hatching did not clearly vary with treatment and group size, either in interaction (−1.42 [−7.582, 4.741]; *P* = 0.651; Figure 2c ; Supplementary Table S8) or as single terms (treatment: −1.969 [−9.523, 5.585]; *P* = 0.68; Figure 2D; group size: 5.002 [−2.02, 12.02]; *P* = 0.116; Supplementary Table S8), but statistical power to detect effects was low due to the limited sample of nestlings that hatched (*N* = 56), and resulting confidence intervals were wide (Figure 2C,D).

## DISCUSSION

Here, we experimentally increased perceived predator presence to study the interactive effects of predation risk and the number of helpers on maternal allocation and reproductive output in a cooperatively breeding species. We predicted that predator-exposed breeding females, and particularly the ones in smaller groups, would reduce resource allocation to reproduction more sharply and have lower reproductive success than the ones in the control treatment. Our results showed that eggs laid in the predator treatment had lighter yolks, specifically the third-laid eggs (the ones collected for this measurement). However, this effect was not clearly conditional on the females’ group size, suggesting that having helpers did not mitigate the observed predator-playback effects on maternal allocation. Offspring in the predator-exposed treatment had a considerably lower mean hatching probability, but this effect was not statistically clear, and again, was not found to vary with breeding group size. In contrast to other studies, the predator treatment did not evidently affect clutch size, egg mass, or egg corticosterone concentration, and fledging success from hatching was also not evidently different between treatments. Even though predator-call playbacks did not generally affect the maternal allocation proxies measured, the detected predator-induced reductions in yolk mass could have consequences for embryonic and post-hatching offspring development.

Previous empirical work investigating how predators influence maternal allocation to reproduction has shown that females often lay less eggs per clutch in response to higher perceived predation risk (predation risk to offspring: Thomson et al. 2006; see review in Martin and Briskie 2009; but see Coslovsky and Richner 2011; predation risk to adults and offspring: Zanette et al. 2011; Hua et al. 2014). Sociable weavers have a relatively high adult survival (Paquet et al. 2015), and lay small but multiple clutches within seasons (generally two to four eggs; Fortuna et al. 2021), which suggests that their lifetime reproductive output is maximized by favoring own survival over fecundity (Erikstad et al. 1998; Ghalambor and Martin 2001). We therefore expected that females perceiving higher predation risk would reduce the number of eggs laid, since feeding rates significantly increase with the number of chicks (Covas et al. 2008; Ferreira 2015), which can consequently increase their exposure to predators. Here, however, we found no evidence that clutch size was different between playback treatments, even though in sociable weavers, there seems to be plasticity in clutch size in relation to other environmental factors, particularly rainfall levels and the risk of nest predation (Fortuna et al. 2021). Moreover, we found no evidence that the predation risk manipulation led to changes in maternal allocation to egg mass (but see Zanette et al. 2011; Hua et al. 2014). Yet, this result concurs with previous findings in sociable weavers showing limited egg mass variation in relation to environmental variables such as predation risk (Fortuna et al. 2021).

On the other hand, females in the predator-playback treatment laid eggs with lighter yolks, representing a mean difference of 0.02 g, that is, a 4% reduction in the yolk mass of third-laid eggs. Yolks contain the major source of nutrients and energy for embryo development (Carey 1996), and these reserves can be used for several days after hatching (Williams 1994). Therefore, yolk mass reductions may correspond to a decrease in the amount of vital nutritional resources available to the developing offspring, which can have detrimental effects on their survival (McGraw et al. 2005; Mentesana et al. 2021; see below). Nevertheless, we only collected the third-laid egg of the clutches to measure the yolk mass. Hence, we cannot determine whether this adjustment was similar for the remaining eggs or exclusive to later-laid ones, and previous investigations in this population suggest only ca. 20–25% repeatability in yolk mass within clutches (Fortuna et al. 2023). Nevertheless, a decrease in yolk mass—generally or specifically in later-laid eggs—might represent a reduced female investment in reproduction, which may lead to brood reduction. However, it might also be a more ‘reversible’ strategy than reducing the number of eggs laid, if conditions improve later, as a low access to nutrients during development and at hatching could be compensated by increased postnatal care (see Fontaine and Martin 2006). Effects of predation risk on maternal allocation to yolk mass have not received much attention in previous investigations. Some exceptions are studies on great tits *Parus major* (Coslovsky et al. 2012) and pied flycatchers *Ficedula hypoleuca* (Morosinotto et al. 2013), where predator-exposed females were found to lay eggs with lower testosterone or higher immunoglobulin levels, respectively, but no differences in yolk mass were detected.

The lighter egg yolks laid by predator-exposed females could arise from different non-exclusive mechanisms. First, predator-call playbacks were performed before egg laying, which may have affected females’ foraging behavior (Lima and Dill 1990; Abbey-Lee et al. 2016) and hence the amount of nutrients available to allocate to offspring (Blount et al. 2004). Additionally, this reduction may represent energy savings for the female, which could be used for future reproduction once conditions improve (Stearns 1992; Carey 1996). Future assessments on whether yolk mass varies with females’ condition in this species could help clarify the mechanism leading to the differences observed here. Furthermore, predator calls may have been a stressor for females and thus increase their plasma circulating corticosterone (Clinchy et al. 2013), which has been found to cause reductions in egg mass and yolk mass in captive birds (Henriksen et al. 2011b). However, yolk corticosterone concentration was not evidently affected by the predation treatment. Even though there are reports of increased corticosterone in eggs of females exposed to predators in some species (Saino et al. 2005), other investigations suggest that the transfer of circulating plasma corticosterone into eggs can be low (Rettenbacher et al. 2005), which may explain why we did not detect differences in egg corticosterone levels between treatments. Given the overall modest effects on egg allocation found here, future experimental studies in this species might benefit from extending the predator-presence exposure to cover several breeding attempts within a season (Zanette et al. 2011) and adding a visual cue, such as a predator decoy.

Regarding offspring survival, our results showed a markedly lower mean hatching probability for eggs laid by females exposed to the predator-call playback, with a mean predicted probability for an egg to hatch in the predation treatment of only 3%, contrasting with 62% in the control treatment, although the statistical differences between treatments were not clear, and model predictions showed large confidence intervals (Figure 2B). This trend concurs with existing literature showing that hatching failure increases in higher predation risk environments (Zanette et al. 2011; Hua et al. 2014), which may be explained by changes in egg quality and composition due to foraging inefficiency or stress levels of breeders (Scheuerlein et al. 2001), and/or by antipredator behavioral responses during the incubation period (Ghalambor and Martin 2000; Zanette et al. 2011). Studies on zebra finches *Taeniopygia guttata* and great tits showed that eggs richer in yolk carotenoids and yolk fatty acids were associated with higher hatching and fledging success (McGraw et al. 2005; Mentesana et al. 2021), which suggests a possible link between the reduced yolk mass and hatching failure observed here. A second (non-exclusive) explanation could be related to the effects of the predator vocalizations on the breeding group behavior at the egg stage, since we only stopped the playbacks when the first egg at each colony hatched. Predator-call playbacks could, therefore, have affected the number of visits of breeders and helpers to the nest (Ghalambor and Martin 2000; Zanette et al. 2011; Santema et al. 2021), which may lead to a decrease in nest attentiveness and protection or to differences in the incubation process. To explore this possibility, we tested treatment effects on visit rates during incubation, but did not find evidence that the number of visits to the nest differed between treatments (see Supplementary Table S9 and Supplementray Figure S2). This however does not rule out that other antipredator behavioral responses at the egg-stage led to lower hatching success, such as clutch reduction by egg ejection (Lobato et al. 2006). After hatching, predator effects on survival to fledging were even less evident, which may indicate that postnatal care compensated for prenatal predator-playback effects on egg composition once offspring hatched. However, treatment effects on fledging probability are likely hard to detect here, as only a small percentage of the offspring that hatched during the experiment survived to fledging, and we may, therefore, lack statistical power to find clear statistical differences between treatments. Overall, these results open interesting questions for future studies on how high predation risk for breeding females may affect their offspring phenotype and survival. Specifically, assessing predator effects on allocation to yolk mass using noninvasive methods (e.g., Ardia et al. 2006) could allow directly assessing whether reduced yolk mass during development negatively affects hatching success but also whether it can lead to changes in offspring phenotype later in life that could be advantageous in high-risk environments (Chin et al. 2009; Coslovsky and Richner 2011; Morales et al. 2018). For instance, reductions in yolk mass, with no corresponding change in egg mass, could represent a higher proportion of albumen in the eggs, which is in turn suggested to accelerate offspring’s embryonic development and post-hatching growth (Ferrari et al. 2006; Willems et al. 2014, 2015).

Importantly, we did not detect interactive effects of breeding group size and predator-induced changes in maternal allocation to eggs. We expected the number of helpers to mitigate predator-induced effects on females’ behavior, condition, stress levels, and reproductive success (Sorato et al. 2012; Hua et al. 2014). Furthermore, as the number of helpers during rearing seems predictable at the time of laying (Paquet et al. 2016; Ferreira et al. 2020), this could allow females to adjust prenatal reproductive investment in relation to environmental cues on helper presence and predation risk (Stearns 1992; Mousseau and Fox 1998; Sheriff and Love 2013; Morales et al. 2018). Instead, we found that the predator treatment similarly affected females regardless of their group size, which suggests that females with more helpers may not be at an advantage particularly under high predation risk environments. This corroborates previous results in this species suggesting lack of helper effects on egg mass and clutch size, either in general or in interaction with climatic conditions and nest predation risk (Fortuna et al. 2021; but see helper effects on egg content in Fortuna et al. 2023). In sociable weavers, it has never been investigated whether predation risk decreases in larger breeding groups, and this would help clarify whether group size confers protection or other advantages to individuals before and during rearing (Sorato et al. 2012). There is, nonetheless, some indication that individuals in larger colonies have higher survival, which may be explained by increased protection from predators when living and foraging in colonial groups (Brown et al. 2003). It is thus possible that even though females with more helpers benefit from reduced parental care and have advantageous associations within breeding groups while foraging and roosting, these effects are small when compared to colonial-living benefits (Angulo et al. 2018), here experienced both by control and predator-exposed females. The combined role of colony sizes and breeding group sizes should, therefore, be considered for future investigations in colonial cooperatively breeding birds. Overall, more studies in this and other species, across years and with substantial sample sizes (e.g., Langmore et al. 2016; Fortuna et al. 2021), are needed for a better understanding of the interplay between group breeding and predator effects, or other environmental cues, on reproductive investment and success.

## CONCLUSION

This study showed that females exposed to predator-call playbacks laid eggs with lighter yolks but were not found to reduce egg or clutch size, and breeding group sizes did not seem to buffer predator-induced changes in maternal allocation. Reductions in yolk mass may have fitness consequences for mothers and their offspring, as these may represent energy savings for the female but negatively affect their reproductive output. The interplay between group breeding and predator effects on maternal allocation and its consequences for offspring phenotype and survival remain largely unexplored in cooperative breeders.

## Supplementary Material

arae010_suppl_Supplementary_Material

## Data Availability

Analyses reported in this article can be reproduced using the data provided by Fortuna et al. (2024).
