# Supplementary material for: Interplay of cooperative breeding and predation risk on egg allocation and reproductive output
Source: Behav Ecol. 2024 Feb 28;35(2):arae010. doi: 10.1093/beheco/arae010 (PMC10939053; doi:10.1093/beheco/arae010)
Supplement: arae010_suppl_Supplementary_Material [file arae010_suppl_supplementary_material.docx]

# Supplementary material to:

# Interplay of cooperative breeding and predation risk on egg allocation and reproductive output

**Authors:** Rita Fortuna^a,b,c,d*^, Rita Covas^a,c,e^, Pietro B. D’Amelio^f,g^ Liliana R. Silva^a,c^, Charline Parenteau^h^, Louis Bliard^i^, Fanny Rybak^g^ , Claire Doutrelant^e,f^, and Matthieu Paquet^j,k^

**Affiliations:**

^a^ CIBIO, Centro de Investigação em Biodiversidade e Recursos Genéticos, InBIO Laboratório Associado, Campus de Vairão, Universidade do Porto, Vairão, Portugal

^b^ Departamento de Biologia, Faculdade de Ciências, Universidade do Porto, Porto, Portugal

^c^ BIOPOLIS Program in Genomics, Biodiversity and Land Planning, CIBIO, Campus de Vairão, Vairão, Portugal

^d^ Centre for Biodiversity Dynamics, Institutt for Biologi, NTNU, Norway

^e^ FitzPatrick Institute, DST-NRF Centre of Excellence, University of Cape Town, Cape Town, South Africa

^f^ CEFE, Univ Montpellier, CNRS, EPHE, IRD, Montpellier, France

^g^ Université Paris-Saclay, CNRS, Institut des Neurosciences Paris-Saclay, 91400, Saclay, France

^h^ Centre d’Etudes Biologiques de Chizé, CNRS-La Rochelle Université, Villiers-en-Bois, France

^i^ University of Zurich, Department of Evolutionary Biology & Environmental Studies, Winterthurerstrasse 190, CH-8057 Zurich, Switzerland

^j^ Institute of Mathematics, University of Bordeaux, Talence, France

^k^ Theoretical and Experimental Ecology Station (SETE), CNRS, Moulis, France

**Corresponding author:** [rita.fortuna@ntnu.no](mailto:rita.fortuna@ntnu.no)

**Data and code availability:** Analyses reported in this article can be reproduced using the data and code provided in <https://doi.org/10.5061/dryad.rjdfn2zkk>.


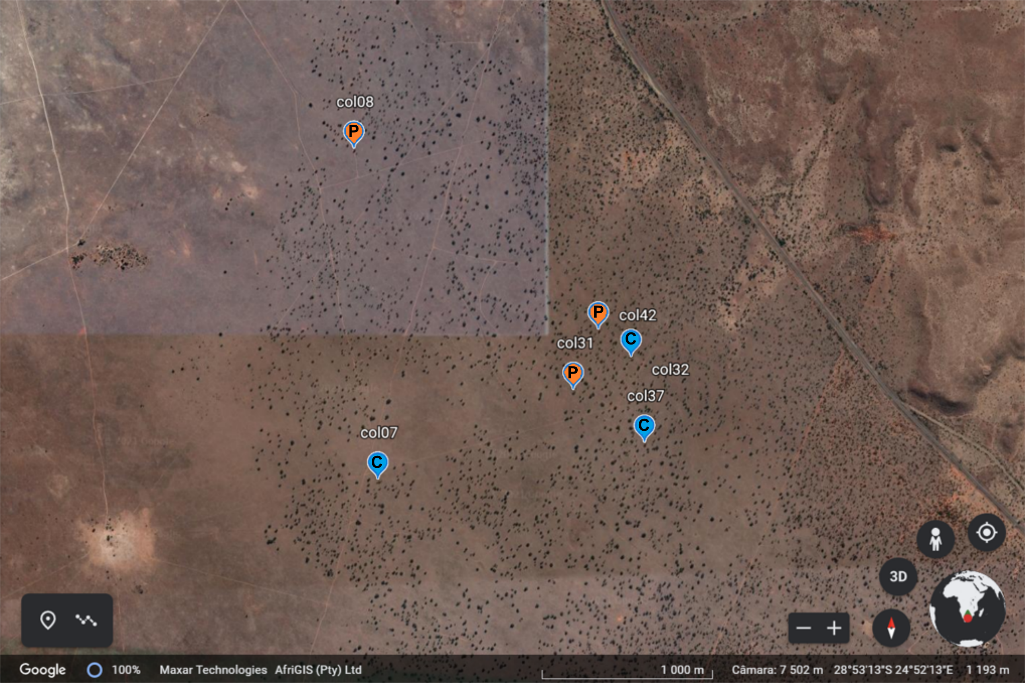


**Figure S1.** Distribution of control (C - blue) and predator-exposed (P - orange) colonies in the study area.

**Table S1.** Results from GLMM on effect of playback treatment and group size (mean=3.7; sd=1.5) on **clutch size** (N=84). Estimates, confidence intervals and significance test results are presented. Reference level for “Treatment” is control.

| **Fixed effect** | **Estimate** | **Std. error** | **t value** | **2.5%** | **97.5%** | **Chisq** | **Df** | **Pr(>Chisq)** |
| --- | --- | --- | --- | --- | --- | --- | --- | --- |
| (Intercept) | 3.608 | 0.17 | 21.243 | 21.243 | 3.288 | - | - | - |
| Group size x Treatment | 0.158 | 0.143 | 1.109 | 1.109 | -0.125 | 1.23 | 1 | 0.267 |
| Group size | -0.128 | 0.109 | -1.174 | -1.174 | -0.335 | 0.258 | 1 | 0.611 |
| Treatment | -0.117 | 0.243 | -0.481 | -0.481 | -0.568 | 0.236 | 1 | 0.627 |
| **Random effect** | **Variance** |  |  |  | |  |  |  |
| Colony ID (N=6) | 0.058 |  |  |  | |  |  |  |
| Nest ID (N=76) | 0.037 |  |  |  | |  |  |  |
| Residual | 0.352 |  |  |  | |  |  |  |

**Table S2.** Results from GLMM on effect of playback treatment and group size (mean=3.7; sd=1.5) on **egg mass** (g; N=257). Estimates, confidence intervals and significance test results are presented. Reference level for “Treatment” is control.

| **Fixed effect** | **Estimate** | **Std. error** | **t value** | **2.5%** | **97.5%** | **Chisq** | **Df** | **Pr(>Chisq)** |
| --- | --- | --- | --- | --- | --- | --- | --- | --- |
| (Intercept) | 2.57 | 0.029 | 87.785 | 2.513 | 2.627 | - | - | - |
| Group size x Treatment | 0.034 | 0.038 | 0.883 | -0.041 | 0.109 | 0.78 | 1 | 0.377 |
| Group size | -0.044 | 0.028 | -1.555 | -0.099 | 0.011 | 1.768 | 1 | 0.184 |
| Treatment | -0.036 | 0.041 | -0.863 | -0.123 | 0.044 | 0.773 | 1 | 0.379 |
| **Random effect** | **Variance** |  |  |  | |  |  |  |
| Colony ID (N=6) | 0 |  |  |  | |  |  |  |
| Nest ID (N=71) | 0.026 |  |  |  | |  |  |  |
| Clutch ID (N=77) | 0 |  |  |  | |  |  |  |
| Residual | 0.014 |  |  |  | |  |  |  |

**Table S3.** Results from GLMM on effect of playback treatment and group size (mean=3.7; sd=1.4) on **yolk mass** (g; N=68). Estimates, confidence intervals and significance test results are presented. Statistically significant results are in bold. Reference level for “Treatment” is control.

| **Fixed effect** | **Estimate** | **Std. error** | **t value** | **2.5%** | **97.5%** | **Chisq** | **Df** | **Pr(>Chisq)** |
| --- | --- | --- | --- | --- | --- | --- | --- | --- |
| (Intercept) | 0.521 | 0.008 | 66.164 | 0.506 | 0.536 | - | - | - |
| Group size x Treatment | -0.018 | 0.011 | -1.606 | -0.04 | 0.003 | 2.58 | 1 | 0.108 |
| Group size | 0.015 | 0.008 | 1.77 | -0.001 | 0.031 | 0.732 | 1 | 0.392 |
| **Treatment** | **-0.023** | **0.011** | **-2** | **-0.044** | **-0.001** | **4.409** | **1** | **0.036** |
| **Egg mass** | **0.03** | **0.006** | **5.127** | **0.019** | **0.041** | **26.287** | **1** | **<0.001** |
| **Random effect** | **Variance** |  |  |  | |  |  |  |
| Colony ID (N=6) | 0 |  |  |  | |  |  |  |
| Nest ID (N=66) | 0.001 |  |  |  | |  |  |  |
| Residual | 0.001 |  |  |  | |  |  |  |

**Table S4.** Results from GLMM on effect of playback treatment and (mean=3.7; sd=1.4) on **yolk mass** (g; N=68) excluding egg mass from model (i.e.: absolute yolk mass change). Estimates, confidence intervals and significance test results are presented. Statistically significant results are in bold. Reference level for “Treatment” is control.

| **Fixed effect** | **Estimate** | **Std. error** | **t value** | **2.5%** | **97.5%** | **Chisq** | **Df** | **Pr(>Chisq)** |
| --- | --- | --- | --- | --- | --- | --- | --- | --- |
| (Intercept) | 0.527 | 0.009 | 57.07 | 0.509 | 0.545 | - | - | - |
| Group size x Treatment | -0.008 | 0.013 | -0.573 | -0.033 | 0.018 | 0.329 | 1 | 0.566 |
| Group size | 0.005 | 0.01 | 0.549 | -0.014 | 0.024 | 0.035 | 1 | 0.852 |
| **Treatment** | **-0.035** | **0.013** | **-2.622** | **-0.06** | **-0.009** | **6.971** | **1** | **0.008** |
| **Random effect** | **Variance** |  |  |  | |  |  |  |
| Colony ID (N=6) | 0 |  |  |  | |  |  |  |
| Nest ID (N=66) | 0.002 |  |  |  | |  |  |  |
| Residual | 0.0008 |  |  |  | |  |  |  |

**Table S5.** Results from GLMM on effect of playback treatment and group size (mean=3.7; sd=1.4) on **yolk corticosterone** concentration (pg/mg; N=68). Estimates, confidence intervals and significance test results are presented. Reference level for “Treatment” is control.

| **Fixed effect** | **Estimate** | **Std. error** | **t value** | **2.5%** | **97.5%** | **Chisq** | **Df** | **Pr(>Chisq)** |
| --- | --- | --- | --- | --- | --- | --- | --- | --- |
| (Intercept) | 4.946 | 0.134 | 36.939 | 4.738 | 5.168 | - | - | - |
| Group size x Treatment | 0.26 | 0.159 | 1.643 | -0.069 | 0.546 | 2.698 | 1 | 0.1 |
| Group size | -0.129 | 0.118 | -1.093 | -0.34 | 0.118 | 0.038 | 1 | 0.845 |
| Treatment | -0.176 | 0.191 | -0.919 | -0.501 | 0.106 | 0.829 | 1 | 0.362 |
| **Random effect** | **Variance** |  |  |  | |  |  |  |
| Colony ID (N=6) | 0.017 |  |  |  | |  |  |  |
| Nest ID (N=66) | 0 |  |  |  | |  |  |  |
| Residual | 0.407 |  |  |  | |  |  |  |

**Table S6.** Results from GLMM on effect of playback treatment and group size (mean=3.7; sd=1.4) on **yolk corticosterone** concentration (pg/mg; N=68) accounting for egg and yolk mass (i.e., absolute change in yolk corticosterone). Estimates, confidence intervals and significance test results are presented. Reference level for “Treatment” is control.

| **Fixed effect** | **Estimate** | **Std. error** | **t value** | **2.5%** | **97.5%** | **Chisq** | **Df** | **Pr(>Chisq)** |
| --- | --- | --- | --- | --- | --- | --- | --- | --- |
| (Intercept) | 4.991 | 0.136 | 36.622 | 4.736 | 5.239 | - | - | - |
| Group size x Treatment | 0.211 | 0.162 | 1.302 | -0.12 | 0.507 | 1.696 | 1 | 0.193 |
| Group size | -0.09 | 0.122 | -0.736 | -0.313 | 0.16 | 0.165 | 1 | 0.685 |
| Treatment | -0.269 | 0.199 | -1.356 | -0.628 | 0.105 | 1.854 | 1 | 0.173 |
| Egg mass | 0.063 | 0.096 | 0.658 | -0.124 | 0.244 | 0.433 | 1 | 0.51 |
| Yolk mass | -0.188 | 0.097 | -1.942 | -0.374 | -0.001 | 3.771 | 1 | 0.052 |
| **Random effect** | **Variance** |  |  |  | |  |  |  |
| Colony ID (N=6) | 0.019 |  |  |  | |  |  |  |
| Nest ID (N=66) | 0 |  |  |  | |  |  |  |
| Residual | 0.394 |  |  |  | |  |  |  |

**Table S7.** Results from GLMM on effect of playback treatment and group size (mean=3.7; sd=1.6) on **egg** **hatching probability** (g; N=141). Estimates (log-odds), confidence intervals and significance test results are presented. Reference level for “Treatment” is control.

| **Fixed effect** | **Estimate** | **Std. error** | **Z value** | **2.5%** | **97.5%** | **Chisq** | **Df** | **Pr(>Chisq)** |
| --- | --- | --- | --- | --- | --- | --- | --- | --- |
| (Intercept) | 0.51 | 1.16 | 0.439 | -1.763 | 2.782 | - | - | - |
| Group size x Treatment | 0.423 | 1.548 | 0.273 | -2.611 | 3.457 | 0.075 | 1 | 0.785 |
| Group size | 0.249 | 1.249 | 0.199 | -2.199 | 2.697 | 0.472 | 1 | 0.492 |
| Treatment | -4.115 | 2.236 | -1.841 | -8.497 | 0.267 | 3.392 | 1 | 0.066 |
| **Random effect** | **Variance** |  |  |  | |  |  |  |
| Colony ID (N=6) | 0 |  |  |  | |  |  |  |
| Nest ID (N=53) | 18.82 |  |  |  | |  |  |  |
| Clutch ID (N=55) | 0 |  |  |  | |  |  |  |

**Table S8.** Results from GLMM on effect of playback treatment and group size (mean=3.9; sd=1.7) on **fledging probability after hatching** (g; N=56). Estimates (log-odds), confidence intervals and significance test results are presented. Reference level for “Treatment” is control.

| **Fixed effect** | **Estimate** | **Std. error** | **Z value** | **2.5%** | **97.5%** | **Chisq** | **Df** | **Pr(>Chisq)** |
| --- | --- | --- | --- | --- | --- | --- | --- | --- |
| (Intercept) | -1.323 | 2.751 | -0.481 | -6.716 | 4.069 | - | - | - |
| Group size x Treatment | -1.42 | 3.144 | -0.452 | -7.582 | 4.741 | 0.204 | 1 | 0.651 |
| Group size | 5.002 | 3.583 | 1.396 | -2.02 | 12.025 | 2.467 | 1 | 0.116 |
| Treatment | -1.969 | 3.854 | -0.511 | -9.523 | 5.585 | 0.17 | 1 | 0.68 |
| **Random effect** | **Variance** |  |  |  | |  |  |  |
| Colony ID (N=6) | 0 |  |  |  | |  |  |  |
| Clutch ID (N=26) | 73.14 |  |  |  | |  |  |  |

**Nest visit rate during incubation**

To test if the playback treatment affected nest visit rates during incubation, we built a LMM with nest visit rate — number of visits by breeding group members divided by total time of recording in minutes — as a dependent variable and treatment, group size, and day of incubation as independent variables. Treatment and day of incubation were included as binary factors (respectively, 0 for control, 1 for predator-call playbacks; videos recorded between “days 2-4” or “days 8-10” of incubation; see Methods). We also controlled for non-independence by fitting clutch identity, nest identity and colony identity as random terms. Analyses were conducted using a similar approach as described for the other models in the Methods.

**Table S9.** Results from LMM on effect of treatment, group size (mean=3.9; sd=1.5) and day of incubation on **nest visit rate** (visits/minute; N=103). Estimates, confidence intervals and significance test results are presented. Reference level for “Treatment” is control and for day of incubation is “days 2-4”).

| **Fixed effect** | **Estimate** | **Std. error** | **Z value** | **2.5%** | **97.5%** | **Chisq** | **Df** | **Pr(>Chisq)** |
| --- | --- | --- | --- | --- | --- | --- | --- | --- |
| (Intercept) | 0.162 | 0.01 | 16.343 | 0.143 | 0.181 | - | - | - |
| Treatment | 0.014 | 0.013 | 1.07 | -0.011 | 0.039 | 1.145 | 1 | 0.285 |
| Group size | 0.012 | 0.007 | 1.813 | -0.001 | 0.025 | 3.287 | 1 | 0.07 |
| Day of incubation | -0.006 | 0.009 | -0.749 | -0.024 | 0.01 | 0.561 | 1 | 0.454 |
| **Random effect** | **Variance** |  |  |  | |  |  |  |
| Colony ID (N=6) | 0 |  |  |  | |  |  |  |
| Nest ID (N=65) | 0 |  |  |  | |  |  |  |
| Clutch ID (N=67) | 0.001 |  |  |  | |  |  |  |


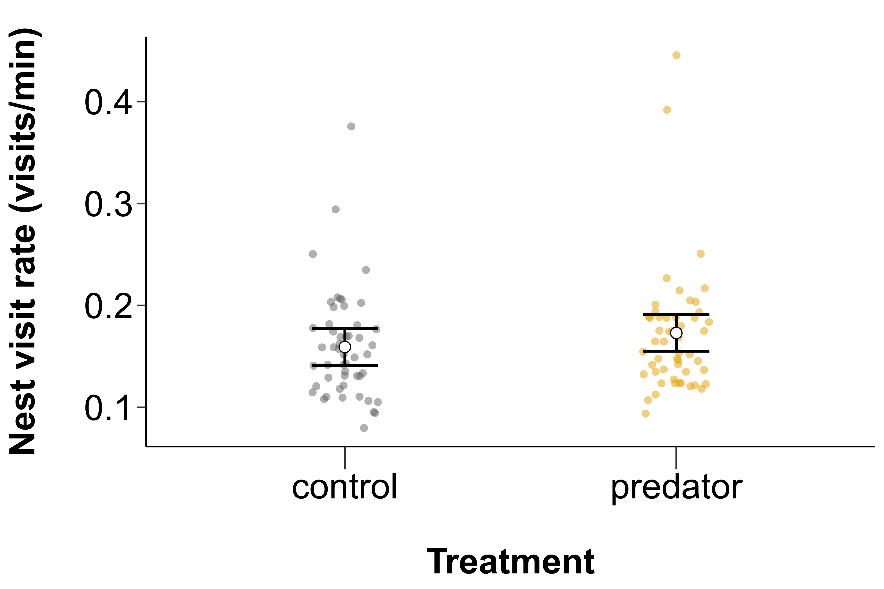


**Figure S2.** Predicted effect of the treatment (control playback – grey points – and predator-call playback – orange points) on nest visit rate during incubation (visits/minute). Circles show mean predicted effects, bars show confidence intervals and points show values for each clutch.
